# Retraction: vB-ApyS-JF1, the first *Trueperella pyogenes* phage, shows potential as an alternative treatment strategy for *Trueperella pyogenes* infections

**DOI:** 10.3389/fmicb.2023.1334746

**Published:** 2023-12-01

**Authors:** Frontiers Editorial Office

Following publication, the authors contacted the Editorial Office to request the retraction of the cited article, stating that further analysis of phage vB-ApyS-JF1 sequencing data discovered contamination with *Mammaliicoccus sciuri* DNA instead of *Trueperella pyogenes*. The conclusions reported in the article are no longer supported by the analyses; therefore, the article has been retracted.

This retraction was approved by the Chief Editors of Frontiers of Microbiology and the Chief Executive Editor of Frontiers. The authors agree to this retraction.

